# Coincidence Anticipation Timing Performance during an Acute Bout of Brisk Walking in Older Adults: Effect of Stimulus Speed

**DOI:** 10.1155/2015/210213

**Published:** 2015-08-31

**Authors:** Michael J. Duncan, Michelle Stanley, Mike Smith, Michael J. Price, Sheila Leddington Wright

**Affiliations:** Centre for Applied Biological and Exercise Sciences, Coventry University, Priory Street, Coventry CV1 5FB, UK

## Abstract

This study examined coincidence anticipation timing (CAT) performance at slow and fast stimulus speeds before, during, and after an acute bout of walking in adults aged 60–76 years. Results from a series of repeated measures ANOVAs indicated significant rest versus exercise × stimulus speed × time interactions for absolute and variable errors (both *P* = 0.0001) whereby absolute and variable error scores, when stimulus speed was slow, improved as the duration of exercise increased. When stimulus speed was fast there were significantly greater absolute and variable errors at 18 minutes of the walking bout. There was also greater error at 18 minutes during walking compared to rest. These results suggest that, in a task involving walking and CAT, stimulus speeds plays an important role; specifically walking (exercise) enhances CAT performance at slow stimulus speeds but reduces CAT performance at fast stimulus speeds. The implications are that in everyday situations, where events require dual-task responses to be made at different speeds, for example, walking on the pavement whilst avoiding a crowd, compared to crossing a busy road, an understanding of how different stimulus speeds influence dual-task performance is extremely important, particularly in the older adult population.

## 1. Introduction

In everyday life, irrespective of age, it is common that in a primary task such as walking the person will also engage in a secondary task involving some aspects of cognitive, perceptual, or motor control [1]. While walking is considered a more automated task in younger adults, it is already known that young adults adopt a slower walking speed under dual-task conditions [2]. Consequently, dual-task conditions pose an interesting paradigm for understanding the effect of walking (acute exercise) in older adults. Indeed, Woollacott and Shumway-Cook [3] postulated that walking becomes a less automated motor pattern with increasing age in adults over 60 years old as greater attentional resources are directed to ensuring gait is maintained.

Recent research has also suggested that in dual-task processing paradigms, where the primary task involves locomotion, performance of a secondary visual task is significantly impaired in older adults [1, 4, 5]. However, few studies have examined this empirically [6] with Beurskens and Bock [1] suggesting that when a visual secondary task is added to walking, the need to manage two streams of visual information concurrently (one related to walking and the other to the secondary task) exceeds the capability of an ageing prefrontal cortex resulting in a decrease in performance on one or both tasks.

Metaregression analysis [7] has identified that the effect of exercise on cognitive performance in adults is equivocal [8–13]. This is partly because most studies have examined cognitive performance on exercise cessation rather than during exercise whilst at the same time making conclusions regarding dual-task effects where exercise and cognitive performance are performed at the same time. In addition to the time of measurement, Lambourne and Tomporowski [7] identified that few studies have examined the effect of exercise on cognitive or perceptual performance in older adults. Therefore, it is particularly important to investigate the effect of exercise on secondary task performance in older adults as it has already been shown that there are age related decrease in brain mass, atrophy of the frontal gray matter, and degradation of the cerebral cortex contributing to a reduction in cognitive processing capacity with advancing age [1].

The effect of exercise on cognitive performance is more likely to be of importance in older adults but is however poorly understood. One study that investigated the effects of exercise on cognitive performance in both young and older adults [14] asked participants to perform the Simon task, which is considered a test of executive function [14], whilst cycling (30 minutes at 65% of age predicted maximum heart rate). In the Simon task, participants are asked to respond as quickly and accurately as possible to a relevant feature of a stimulus (the colour) whilst inhibiting an irrelevant feature (the spatial location) of the same stimulus. Joyce et al. [14] noted that, although there were no differences in reaction time between older and younger adults, older adults adopted a more cautious approach to their task resulting in lower error rate compared to their younger peers. Other work by Barella et al. [15] investigated the duration of effects in cognitive performance (Stroop test) following 20-minute treadmill walking at an intensity of 60% heart rate reserve (HRR) in a sample of 40 adults aged 60–90 years. Barella et al. reported an immediate improvement in reaction time performance in the Stroop colour test immediately on exercise cessation but no other improvements or longer-term enhancement of cognitive performance.

One particular facet of cognitive performance, which is related to tasks of daily living, is coincidence anticipation timing (CAT). CAT is the ability to predict the arrival of a moving object at a particular point in space and coordinate a movement response with that arrival [16] and is strongly related to numerous tasks of daily living, particularly where predicting the arrival of a moving object is important, for example, crossing a busy street [17] or walking through a crowd while keeping an eye on another individual [5]. For example, when standing on the pavement of a busy street, the ability to predict when a car will reach (or pass) the place in the road where the pedestrian will cross is important. Likewise, when walking through a crowded street being able to “time” other individuals as they walk towards/around an individual is important in avoiding collisions or intercepting an individual (or moving object). It has been shown that older adults are negatively affected in tasks that have an element of CAT incorporated within them [18, 19] and that at rest the stimulus speed of the anticipation timing task (i.e., how fast the target or “stimulus” moves) may impact on CAT performance [20]. How exercise impacts on CAT and whether stimulus speed of the timing task impacts on any effect of exercise on CAT in older adults are yet to be established. This study sought to advance prior research by examining CAT performance at slow and fast stimulus speeds before, during, and after an acute bout of walking in older adults.

## 2. Materials and Methods

### 2.1. Participants

Following institutional ethics approval informed consent, 16 older adults (aged 60–76 years, age: 65.6 ± 4.1 years, 7 females, 9 males) volunteered to participate. Descriptive data for the sample are presented in Table 1. All participants were habitually physically active (>150 min per week). Participants were excluded if they had any cardiovascular condition or were taking medications such as beta blockers or calcium ion channel blockers. Participants were asked to abstain from exercise for 24 hours prior to each visit and refrain from caffeine on waking each morning of the testing.

### 2.2. Procedures

The study used a repeated-measures design whereby participants undertook 3 visits to the laboratory. During the initial test session each participant was familiarised with the Bassin Anticipation Timer (Model 35575, Lafayette, USA) and given 20 attempts at each of the stimulus speeds used in the study (3 and 8 mph) to familiarise themselves with the test protocol. Resting heart rate (HRrest) was obtained from each participant whilst wearing a heart rate monitor (Polar RS400, Polar Electro Oy, Kempele, Finland), while they lay in a supine position for 10–15 minutes in a quiet room void of visual or auditory distractions. HRrest was recorded and used to calculate 50% age predicted HRR [6] to be used in the subsequent exercise trial. In the following two experimental trials, participants completed measures of CAT before, during, and immediately following a 20-minute passive rest condition or a 20-minute exercise condition. Each visit to the laboratory was separated by at least 72 hours and conditions were counterbalanced. For each condition, CAT measures were taken before, during: at 9 and 18 minutes, and after exercise.

### 2.3. Exercise Condition

In the exercise condition, participants were asked to walk on a treadmill (HP Cosmos Ltd., Germany) for 20 minutes at an intensity of 50% of HRR [6]. In the current study the threshold of 50% HRR was chosen as this is within the spectrum of exercise recommended by the ACSM for enhancement of cardiovascular fitness [21]. A 20-minute exercise duration was chosen as Lambourne and Tomporowski [7] identified this duration as most appropriate to identify any decrement in dual-task performance as a consequence of exercise. Heart rate was monitored throughout each trial. Borg's [22] rating of perceived exertion 6–20 (RPE) scale was used as an adjunct to the monitoring of heart rate. Participants were required to achieve an RPE of 13–15 during the exercise trial. An RPE of 13–15 is considered “light to moderate” intensity activity and commensurate with brisk walking. Walking speed was modified throughout to ensure that HRR remained at 50%, as has been the case in other studies [9, 13]. Walking speeds for the exercise condition, across all participants, were in the range of 4.4–6.5 km/h. This is congruent with reported speeds for “brisk” walking in older adults [23]. There was intratrial variation of approximately 0.5 km/h in walking speed to ensure each participant remained at the required 50% HRR. At 9 minutes and 18 minutes during the exercise condition, participants performed 10 trials on the CAT task at each of the stimulus speeds of the timing task (3 and 8 mph) whilst still walking. The decision to assess CAT at 9 and 18 minutes during each condition was again based on the findings of prior metaregression analysis [7] showing different effect sizes for cognitive tasks performed from 0–10 minutes and 11–20 minutes during exercise. The rationale for the choice of stimulus speeds during the timing task was based on prior work with older adults which used stimulus speeds of 3 and 8 mph as indicative of “slow” and “fast” speeds [20].

### 2.4. Rest Condition

In the rest condition, participants were seated in a quiet room. At 9 minutes and 18 minutes during this condition participants performed 10 trials on the CAT task at each of the stimulus speeds (3 and 8 mph) whilst standing on the treadmill.

### 2.5. The Bassin Anticipation Timer

**Figure 1:**
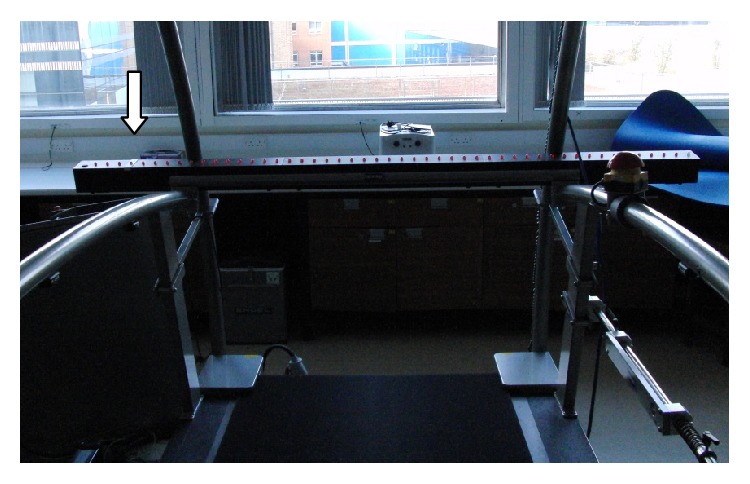
Experimental setup of the Bassin Anticipation Timer. Arrow indicates location of the target light with the motion of the stimulus light moving from right to left.

The Bassin Anticipation Timer was set up horizontally across the front of the treadmill. This enabled participants to complete the CAT trials whilst walking. The mean time to complete the CAT trials was 60 seconds. Three sections of runway (2.24 m) with the system's LED lights facing the participant were used with the runway sections mounted onto the treadmill. None of the lights on the runway were blanked and the target light was light number 13. The experimental setup is depicted in Figure 1. The sequentially lighted LED lamps illuminate in a linear pattern with movement occurring from right to left. For each trial, scores were recorded in milliseconds (ms) and whether the response was early or late. The start and end speeds remained constant at 3 and 8 miles h^−1^ for all trials. To reduce the likelihood that the participant could internally time the trial, cue delay (visual warning system) was set as random on the timer with a minimum delay of 1 second and a maximum delay of 2 seconds. For each trial, the signal was initiated by the experimenter. The participant was asked to press a trigger button, with their dominant hand, as close to the arrival time of the stimulus at the target location as possible. This is congruent with other research which has examined CAT during exercise [13].

### 2.6. Statistical Analysis

The results are expressed as mean and standard deviation (SD). Each participant's raw scores across each of the stimulus speeds were summarised into three error scores as a means of generating the dependent variables. This is consistent with the recognised protocols using CAT scores [9, 13, 17, 24]. The dependent measures were as follows.

#### 2.6.1. Absolute Error

The absolute value of each raw score disregarding whether the response was early or late was calculated. Absolute error provides the best representation of both the individual and combined effects of task characteristics as a whole [17] and as a consequence tends to be the most widely reported CAT outcome variable in the literature [9, 17]. The data for absolute error were however positively skewed (all the values are positive). To correct for skewness, the data set were log transformed as log transforming data in this way has been shown to overcome skewness in previous work [9, 25].

#### 2.6.2. Variable Error

The participant's standard deviation from his mean response; this represents the variability/inconsistency of responses. Similar to absolute error, the data for variable error were skewed and therefore data were log transformed as per previous authors [9, 25].

#### 2.6.3. Constant Error

The temporal interval (milliseconds) between the arrival of the visual stimulus and the end of the participant's motor response. It represents the mean response of an individual and the direction of error (i.e., early or late) [26].

To compare the effect of rest versus exercise on constant error, absolute error, and variable error, a 2 (rest versus exercise) × 2 (stimulus speeds) × 4 (time intervals, before, at 9 min and 18 min during, and after) repeated measures analysis of variance (ANOVA) was employed. Where significant differences were found, Bonferroni post hoc pairwise comparisons were used to determine where the differences lay. Partial eta squared (*η* ^2^) was also used as a measure of effect size. The Statistical Package for Social Sciences (SPSS, Version 20, Chicago, IL, USA) was used for all analysis and statistical significance was set, a priori, at *P* = 0.05.

## 3. Results

Mean ± SE of absolute, constant, and variable errors (secs) at stimulus speeds of 3 and 8 mph before, at 9 minutes and 18 minutes during, and after 20-minute rest or 20-minute walking at 50% HRR is presented in Table 2.

### 3.1. Absolute Error

**Figure 2:**
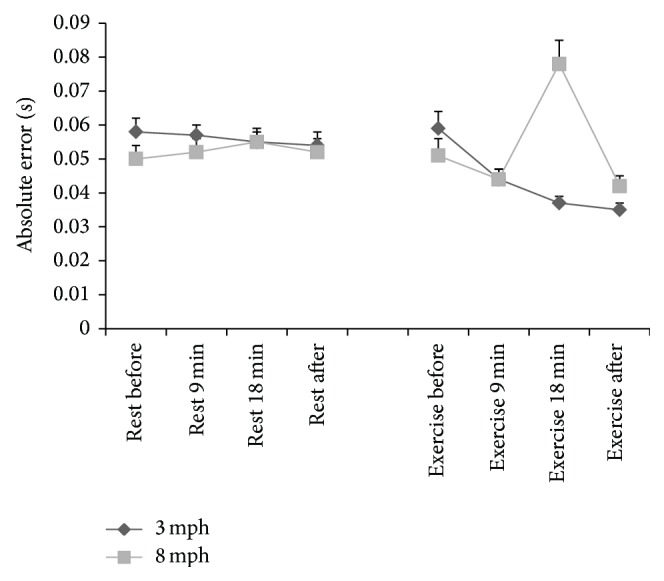
Mean ± SE of absolute error (secs) in rest and exercise conditions, at stimulus speeds of 3 and 8 mph and in rest and exercise conditions.

Absolute error demonstrated a significant rest versus exercise × stimulus speed × time interaction (*P* = 0.0001, partial *η* ^2^ = 0.481, Figure 2). Absolute error at 9 minutes during exercise was significantly lower than rest at stimulus speeds of 3 mph (*P* = 0.006) and 8 mph (*P* = 0.033). For stimulus speed of 3 mph this trend continued with lower absolute error during exercise at 18 min (*P* = 0.0001) and after exercise (*P* = 0.0001) compared to rest. However, when stimulus speed was 8 mph there was significantly smaller absolute error at 9 min during exercise compared to rest (*P* = 0.033). This trend was reversed at the 18 min time point with absolute error scores during rest being significantly greater than during exercise (*P* = 0.003). After exercise, absolute error scores were significantly lower in the exercise condition compared to the rest condition (*P* = 0.014).

### 3.2. Variable Error

**Figure 3:**
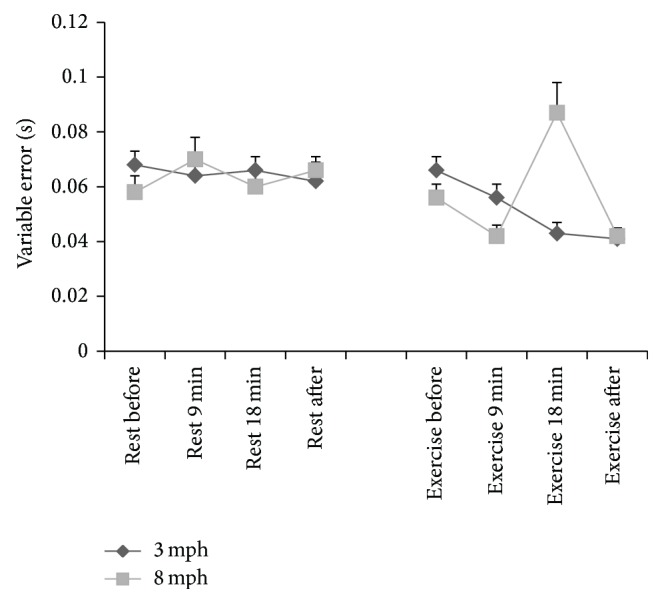
Mean ± SE of variable error (secs) in rest and exercise conditions, at stimulus speeds of 3 and 8 mph and in rest and exercise conditions.

Variable error also revealed a significant rest versus exercise × stimulus speed × time interaction (*P* = 0.0001, partial *η* ^2^ = 0.451, Figure 3). When stimulus speed was 3 mph, variable error was significantly lower during exercise at 18 min (*P* = 0.001) and after exercise (*P* = 0.029) compared to rest. When stimulus speed was 8 mph variable error was significantly lower during exercise compared to rest at 9 min (*P* = 0.018) and after exercise/rest (*P* = 0.0001). However, at 18 min during exercise variable error was significantly higher during exercise compared to rest (*P* = 0.026).

### 3.3. Constant Error

**Figure 4:**
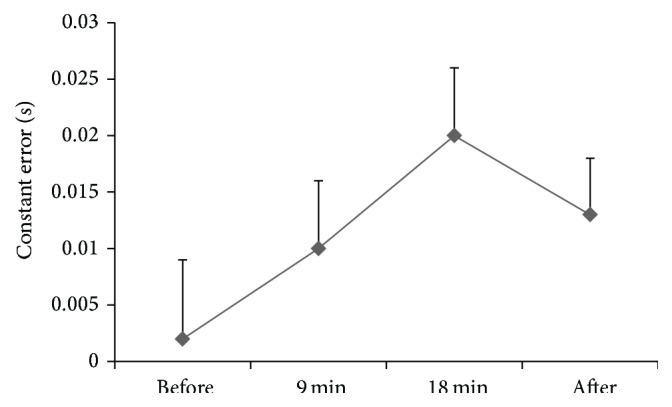
Mean ± SE of the time main effect for constant error (secs).

The repeated measures ANOVA indicated that there were no significant higher-order interactions for constant error. However, there was a significant main effect for time (*P* = 0.0001, partial *η* ^2^ = 0.275, Figure 4). Bonferroni post hoc multiple comparisons indicated that constant error scores were significantly higher at 18 min compared to preexercise/rest (*P* = 0.003) and 9 min (*P* = 0.028).

## 4. Discussion

The purpose of this study was to investigate the effects of different stimulus speeds on CAT performance in older adults before, during, and after exercise in a sample of healthy older adults. The current study suggests that CAT performance was improved during and immediately after exercise (at both 9 minutes and 18 minutes during), compared to rest, but only when stimulus speed was slow (i.e., 3 mph). However, when the stimulus speed of the task was faster (i.e., 8 mph) and consequently more demanding there was a significant increase in absolute and variable errors in the latter half of the 20-minute exercise bout (i.e., poorer performance at 18 minutes during the exercise). The data presented here are novel as no studies to date have examined anticipation timing performance during and after exercise in older adults. Nor has prior work examined any impact of different stimulus speeds on anticipation timing performance in older adults.

The results of the present study add partial support to previous research that has suggested that moderate-intensity exercise results in a significant improvement in performance of cognitive-perceptual, psychomotor, and sport-specific skills [9, 13]. It has also been suggested that moderate-intensity exercise elicits optimal levels of CNS arousal [27, 28] which, among other performance indicators, improves reaction time. Åstrand et al. [29] further add that moderate-intensity exercise is beneficial to performance due to increased blood flow, warming up of the musculature, and increased speed of nerve transmission within the PNS. In the context of an aging brain, it is possible that this increased blood flow and CNS arousal offset the age related reduction in cerebral blood flow associated with poorer cognitive processing capacity with advancing age [30]. However, in the present study, this suggestion only applies where stimulus speed was slow. When the task was more demanding (via faster stimulus speed) there was an increase in both the error and variability of CAT responses at 18 minutes during the exercise bout (primary task).

In some ways this is not surprising as an increase in dual-task costs occurs when there is a need to manage two streams of similar (e.g., visual) information (i.e., when walking and attending to the CAT task in the present study) compared to when managing two tasks requiring different forms of processing (e.g., one visual and one auditory) [4]. Moreover, errors tend to be higher when task difficulty is greater during dual-task situations that both rely on visual processing in some form [4]. In the present study, the increased physiological demand of walking for 20 minutes, combined with the increased demands of the cognitive task, may have resulted in poorer secondary task performance as changes to gait (i.e., slower walking speed; reduced stride length) would not be possible when walking on a treadmill at a set intensity. This is consistent with assertions previously made by Beurskens and Bock [1].

The loss of central neurons and associated synaptic connections occurs with increasing age in older adults [31]. This, in turn, leads to reduced processing speed and consequently a deficit in the ability to process several tasks simultaneously [31]. This may provide an explanation why older adults' cognitive performance was significantly affected in the present study when the stimulus speed was increased to 8 mph. The fact that CAT tasks were performed during walking may also be fundamental to understanding how exercise might impact on situations in which anticipating correctly is of paramount importance, like crossing a busy road, for example [17]. The present study acted on suggestions by Lambourne and Tomporowski [7] that, in order to understand how exercise impacts on other types of skills, cognitive tests must be conducted during the exercise task rather than after exercise. Lambourne and Tomporowski's [7] suggestions are extremely pertinent to the present study as if only the pre- and postexercise data are considered, the participant responses to both the slow and fast stimulus speeds would appear to show an improvement in timing accuracy after exercise. When data are considered as a continuum or before, during, and after exercise, the effect of the dual-task paradigm becomes apparent.

The magnitude of differences seen at this point, either in comparison to rest data or other data points during, before, or after exercise, is larger than that seen in athletes [9] or young adults [13]. They are also more than double the duration reported for timing of catching actions when stimuli are sighted [32]. Consequently, the differences reported here may be considered as meaningful in the context of anticipation timing in a human movement context. Whether this magnitude of change is partly attributable to aging is yet to be established and future studies comparing the responses of younger to older adults would be useful here. Although no studies to date have examined the effect of exercise on anticipation timing responses in older adults, it is interesting to note that Duncan et al. [13] reported a similar trend in CAT responses to fast stimulus speeds in younger adults but only when exercise intensity was considerably high (90% HRR) than in the present study. Collectively, this supports suggestions by Menant et al. [4], Pothier et al. [5], and Beurskens and Bock [1] that older adults have greater difficulty than younger adults in carrying out walking and cognitive tasks simultaneously due to lack of neural plasticity, where cognitive processing can replace automated sensorimotor processing with higher-order functions.

It is unclear why there might be differing results for constant (directional) and absolute (nondirectional) error scores in the present study. This finding is however not uncommon in the CAT literature [9, 13, 17]. The current results would seem to indicate that increased stimulus speed during exercise results in less accuracy in CAT but in a nonsystematic way. It is also possible that, in line with Kahneman's [33] multidimensional allocation of resources theory, responding to faster stimulus speed as primary task duration increased resulted in increased demands of the concurrent activities with a corresponding greater demand on attentional resources and potentially poorer performance. This aligns with research focused on neural plasticity [3], whereby the demand on cognitive resources allocated to maintaining gait in older adults is kept high (by virtue of a set treadmill walking intensity) leaving less resources available for other activities and resulting in poorer secondary task performance when secondary task demand is higher [14]. For example, the prioritization for postural control over cognitive process, where there is a threat to postural stability [34], results in a situation where cognitive-perceptual tasks cannot be completely attended to until the appropriate postural responses have been initiated (or inhibited) [34]. In the present study, where the secondary task was more demanding, the prioritization of maintaining gait during treadmill walking led to a situation where cognitive workaround strategies that would normally replace sensorimotor processing were not as effective due to high resource allocation demands for both the treadmill walking and secondary (CAT) tasks.

Despite the findings presented here, this study is not without limitation. The task employed in the present study required the participant to briskly walk on a treadmill at a standardised exercise intensity and duration. This intensity equates to a brisk walk at a duration equated with health benefit in older adults [34]. This allowed for locomotive control of the primary task demands, as the participants could not alter the resource allocated to the primary task (i.e., reduction in walking speed as a compensatory measure) when they were required to carry out the cognitive tasks. In the current study the motion of the Bassin Timer moved in a linear fashion from right to left in front of the participant. This may arguably have made the task somewhat different to the typical anticipatory movements made in some aspects of daily life (e.g., catching a ball thrown to an individual; negotiating a busy shopping precinct) but may be more akin to anticipatory decisions made when crossing a busy road, for example, where a car may typically move linearly past a pedestrian. From an ecological perspective the instances where brisk walking and CAT tasks occur at the same time in general in older adults' lives are not known and therefore the direct application of the present study to the daily lives of older adults remains equivocal. Future research would be beneficial which incorporates nonlinear motion, tasks that better represent the dual-task conditions experienced by older adults, and a requirement to respond to secondary task demands unexpectedly as these task parameters may pose a greater demand on cognitive processes for an aging brain [1].

Finally, the participants in the present study were all apparently healthy, free from disease, and habitually physically active. Whether the responses presented here are similar in older adults who are not physically active or have a reduced cognitive capacity has yet to be established.

The results of the present study suggest that, in a dual-task processing paradigm involving walking and CAT tasks, the stimulus speeds play an important role for older adults, whereby exercise enhances timing performance when stimulus speed is slow but reduces performance when stimulus speed is fast. These findings may be indicative that where gait needs to be maintained, cognitive workaround strategies that would normally replace sensorimotor processing are not as effective when attentional demands of the secondary task are greater.

## Figures and Tables

**Table 1:** Descriptive data.

	Mean	SD
Age (years)	65.6	4.1
Height (m)	1.68	0.07
Body mass (kg)	74.1	13.5

**Table 2:** Mean (SE) of absolute, constant, and variable errors (secs) at stimulus speeds of 3 and 8 mph before, at 9 min and 18 min during, and after 20-minute rest or 20-minute walking at 50% HRR.

	Rest				Exercise				
	Before	9 min during	18 min during	After	Before	9 min during	18 min during	After	
Absolute error 3 mph (secs) Absolute error 8 mph (secs)	0.05 (0.004) 0.049 (0.003)	0.055 (0.003) 0.054 (0.004)	0.054 (0.002) 0.051 (0.003)	0.053 (0.003) 0.053 (0.003)	0.06 (0.004) 0.051 (0.004)	0.043 (0.003) 0.043 (0.002)	0.036 (0.002) 0.077 (0.006)	0.034 (0.001) 0.041 (0.003)	*F*_3,45_ = 14.385, *P* = 0.0001, partial *η* ^2^ = .481, rest versus exercise × stimulus speed × time interaction

Constant error 3 mph (secs) Constant error 8 mph (secs)	0.008 (0.006) −0.003 (0.007)	0.012 (0.007) 0.007 (0.005)	0.014 (0.006) 0.019 (0.003)	0.013 (0.006) 0.008 (0.007)	0.004 (0.009) 0.002 (0.007)	0.002 (0.007) 0.016 (0.007)	0.015 (0.004) 0.030 (0.01)	0.013 (0.004) 0.015 (0.006)	*F*_3,45_ = 9.949, *P* = 0.001, partial *η* ^2^ = .275, main effect for time (before, during, and after)

Variable error 3 mph (secs) Variable error (secs)	0.069 (0.005) 0.057 (0.005)	0.063 (0.003) 0.067 (0.010)	0.065 (0.004) 0.057 (0.004)	0.062 (0.006) 0.063 (0.005)	0.07 (0.006) 0.056 (0.004)	0.054 (0.004) 0.041 (0.003)	0.042 (0.002) 0.087 (0.01)	0.041 (0.003) 0.041 (0.002)	*F*_3,45_ = 12.735, *P* = 0.0001, partial *η* ^2^ = .459, rest versus exercise × stimulus speed × time interaction
